# Lin28A Regulates Stem-like Properties of Ovarian Cancer Cells by Enriching RAN and HSBP1 mRNA and Up-regulating its Protein Expression

**DOI:** 10.7150/ijbs.43504

**Published:** 2020-04-15

**Authors:** Yancheng Zhong, Lanqin Cao, Haotian Ma, Qian Wang, Pingpin Wei, Juan Yang, Yuqing Mo, Lihua Cao, Cijun Shuai, Shuping Peng

**Affiliations:** 1 NHC Key Laboratory of Carcinogenesis of Hunan Cancer Hospital and the Affiliated Cancer Hospital of Xiangya School of Medicine; School of basic Medical Science, Central South University, Changsha, Hunan 410013, China.; 2 The Key Laboratory of Carcinogenesis and Cancer Invasion of the Chinese Ministry of Education, Xiangya Hospital, Central South University, Changsha, 410078, China.; 3 Hunan Key Laboratory of Non-resolving Inflammation and Cancer, Disease Genome Research Center, the Third Xiangya Hospital, Central South University, Changsha, 410013, China.; 4 The department of gynecology of Xiangya Hospital, Central South University, Changsha, Hunan, China.; 5 Jiangxi University of Science and Technology, Ganzhou, 341000, China; State Key Laboratory of High Performance Complex Manufacturing, Central South University, Changsha, 410083, China.

**Keywords:** Lin28A, RAN, HSBP1, stemness, apoptosis, invasion

## Abstract

Ovarian cancer (OC) is one of the malignant tumors that seriously threaten women's health, with the highest mortality rate in gynecological malignancies. The prognosis of patients with advanced OC is still poor, and the 5-year survival rate is only 20-30%. Therefore, how to improve the early diagnosis rate and therapeutic effect are urgent for patients with OC. In this research, we found that Lin28A can promote the expression of stem cell marker molecules CD133, CD44, OCT4 and Nanog. We later confirmed that Lin28A can enrich the mRNA of ras-related nuclear protein (RAN) and heat shock factor binding protein 1 (HSBP1) through RIP assay, and that Lin28A can regulate their protein expression. We also identified that RAN and HSBP1 are highly expressed in OC tissues, and that they are significantly positively correlated with the expression of Lin28A and negatively correlated with the survival prognosis of OC patients. After stable knockdown of RAN or HSBP1 in OC cells with high expression of Lin28A, the expression of the stem cell marker molecules such as OCT4, CD44 and Nanog are reduced. And after knocking down of RAN or HSBP1 in Lin28A highly expressed OC cells, the survival and invasion of OC cells and tumor size of OC xenograft in nude mice were markedly inhibited and apoptosis was increased. Our data also showed that knock down of RAN or HSBP1 can inhibit the invasion ability of OC cells by decreasing the expression of N-cadherin, Vimentin and promoting the expression of E-cadherin. Meanwhile, knockdown of RAN or HSBP1 induced cell apoptosis by inhibiting the expression of PARP. Our results indicated that Lin28A could regulate the biological behaviors in OC cells through RAN/HSBP1. These findings suggest that Lin28A/RAN/HSBP1 can be used as a marker for diagnosis and prognosis of OC patients, and RAN/HSBP1 may be a potential new target for gene therapy of OC.

## Introduction

Ovarian cancer (OC) is the most lethal of gynecological malignancies, according to ovarian cancer statistics, 14070 patients died of OC in the United States in 2018^1^, ^2^. OC accounts for 3% of the total incidence of gynecological cancer, while is one of the highest mortality gynecological malignancies^3^, ^4^. The main histotypes of OC are epithelial in origin, of which high-grade serous carcinoma accounts for 70%-90%^5^, ^6^. At present, the main treatments for OC patients include surgery, platinum and paclitaxel combined chemotherapy, Cetuximab, a monoclonal antibody that inhibits epidermal growth factor receptor (EGFR)^7^-^9^. The emergence of PARP inhibitors has brought new hope to people, but only 10-20% of patients with OC carry BRCA1/2 mutation^9^-^12^. Although there is some relief for patients without mutation, there are still some patients with no significant effect. The survival rate of OC patients has not well improved, the 5-year survival rate is only 20%-30%^1^, ^13^. Due to the lack of molecular mechanisms of chemoresistance and OC metastasis, conventional treatment is difficult to further improve clinical outcomes. Therefore, it is necessary to further understand the mechanism of recurrence and metastasis of OC in order to develop new diagnostic and therapeutic strategies.

With the increasing research on recurrence and chemoresistance in patients with OC, ovarian cancer stem cells have been receiving attention until now. The researchers who first extracted so-called cancer stem cells from the ascites of OC patients believe that stem cell transformation may be the cause of ovarian cancer, and random events of stem cell transformation eventually lead to increased invasiveness of OC^14^. It has been reported that ALDH1^+^, CD24^+^, CD133^+^, CD117^+^ and CD44^+^ have been considered as markers of OC stem cell^15^, ^16^. Currently, cancer stem cells are defined as a relatively small population of cancer cells that have an ability to undergo lifelong regeneration and differentiation in an asymmetric manner. Cancer stem cells may remain static in the body for a long time and are resistant to conventional chemotherapy and radiation, the resistance and recurrence of OC may be related to OC stem cells^17^.

RNA-binding protein Lin28A (also known as Lin28) is a key factor in the maintenance of pluripotency of embryonic stem cells^18^, ^19^. Numerous studies have pointed out that the highly expressed Lin28A is association to advanced human malignancies, and Lin28A mainly plays an important role in promoting cancer development through let-7^20^-^23^. However, as an RNA-binding protein, Lin28A regulates not only the function of miRNA, but also the function of mRNA^23^. Our previous studies indicate that Lin28A can promote the proliferation, invasion of OC cells and inhibit the apoptosis of OC cells by promoting the expression of the interacting protein ROCK2^23^, ^25^.

And currently, we found that highly expressed Lin28A not only affects the ratio of side population but also promotes the expression of OC stem cell marker molecules CD133, CD44, OCT4 and Nanog. Mechanistically, we found that Lin28A can enrich the mRNA of RAN and HSBP1 and up-regulate the expression of their protein levels. We also identified that Lin28A, RAN and HSBP1 are highly expressed in OC tissues, and there is a significant positive correlation between the expression of RAN/HSBP1 and Lin28A in OC tissues. After knocking down the expression of RAN/HSBP1 in OC cells with highly expressed Lin28A, the survival and invasion were significantly inhibited and apoptosis was increased in OC cells and OC xenograft in nude mice. At the same time, knockdown of RAN or HSBP1 in cells also down-regulated the stem markers of CD44, CD133 and Nanog. Although the detailed mechanism of Lin28A regulation on OC stem cell, invasion, and apoptosis of OC cells need further study, the results indicate that Lin28A can regulate the malignant development of OC cells by binding to the mRNA of RAN/HSBP1 and upregulating their protein levels. In conclusion, our study suggest that highly expressed Lin28A/RAN/HSBP1 can be used as diagnostic and prognostic markers in patients with OC, whereas RAN/HSBP1 may be a potential new target for gene therapy of OC.

## Materials and Methods

### Cell lines and cell culture

The OC epithelial cell line A2780 and PA-1 were purchased from ATCC and the Chinese Academy of Medical Sciences, Institute of Basic Medical Sciences, Beijing Union Medical College Cellular Resource Center, respectively. The above cells have been tested and confirmed to be free of mycoplasma and cross-contamination. A2780 cells were cultured in DMEM medium (Gibco, USA) containing 10% fetal bovine serum (Gibco, USA) and 1% penicillin/ streptomycin (BI, Israel). PA-1 was cultured with MEM medium (Hyclone, USA) with a fetal bovine serum (Gibco, USA) concentration of 10% and 1% penicillin/streptomycin (BI, Israel). Above cell lines were cultured at 37°C and 5% CO^2^.

### Patients and tissue samples

Malignant ovarian cancer tissue samples and benign ovarian tumor tissue samples were obtained from 140 patients who were underwent ovarian surgical resection at the Department of General Surgery of the Xiangya Hospital of Central South University, China, between April 2010 and May 2016. Each patient signed a written informed consent for tissue collection and related molecular analysis.

### Antibodies

The following antibodies are used in immunohistochemistry studies: anti-Lin28A (ab124765, Abcam, USA), anti-RAN (ab53775, Abcam, USA), anti- HSBP1 (ab83247, Abcam, USA), anti-Ki67 (D154094, BBI Life Sciences, China), anti-E-cadherin (20874-1- AP, Proteintech, China). The antibodies used for western blot analysis are as follows: anti RAN (10469- 1-AP, Proteintech, China), anti-HSBP1 (DF8954, Affinit, China), anti-Actin (AC004, ABclonal, China), anti-E-cadherin (20874-1-AP, Proteintech, China), anti-N-cadherin (22018-1-AP, Proteintech, China), anti-Vimentin (10366-1-AP, Proteintech, China), Apoptosis Antibody Sampler Kit (#9915T, CST, USA), Epithelial-Mesenchymal Transition (EMT) Antibody Sampler Kit (#9782, CST, USA).

### Quantitative real-time PCR (qRT-PCR)

Total RNA was isolated using Trizol reagent and reverse transcribed into cDNA. Thereafter, qRT-PCR was used to detect the expression of each molecule using SYBR Premix Ex Taq Kit (B21202, Selleck, China). Actin and tubulin were used as internal control. The primer sequences used for qRT-PCR were shown in Table S1.

### Western blot analysis

Cells were first washed three times with ice-cold PBS, then suspended in RIPA buffer containing a mixture of protease inhibitors and then placed on ice for 15 minutes with shaking 2-3 times. And centrifuge at 13,000 rpm for 15 minutes at 4˚C and transfer the supernatant to a new tube. Then 50 μg of total protein was separated by SDS-PAGE and transferred to a PVDF membrane (Milipore, Germany). The membrane was blocked with 5% milk at room temperature for 1 hour and then incubated with primary antibodies overnight at 4˚C. The next day, the PVDF membrane was incubated with HRP- conjugated anti-rabbit or anti-mouse IgG antibodies according to the isotype of the primary antibodies. Finally, membranes were imaged and analyzed using the Chemi Doc MP System (Bio-Rad, Hercules, CA, USA).

### Immunohistochemical staining

The paraffin tissue slices were heated at 60˚C for 1 hour in incubator, then dewaxed in xylene and rehydrated by gradient ethanol. Then the slices were immersed in 3% peroxide for 20 minutes to prevent endogenous peroxidase activity. The tissue sections were heated until 100˚C to recover the antigen in an autoclave containing antigen retrieval solution for 8 minutes. After cooling to room temperature, the slides were washed 3 times with PBS were blocked with ready-to-use goat serum for 1 hour and then incubated with primary antibody at 4˚C overnight. The next day, after washing three times with PBS, the slides were incubated with the secondary antibody solution at 37˚C for 1 hour and then with DAB. Finally, the sections were counterstained with hematoxylin, dehydrated in a gradient alcohol solution, sealed with neutral gum, and observed under the Optical Microscope (Olympus, Japan)^26^.

### RNA interference of RAN/HSBP1

Specific small interference RNAs (siRNAs) were designed and synthesized by Genepharma (Shanghai, China). The siRNA sequences against RAN were: RAN siRNA#1 (si1), 5'-GGT GAA GCT GAA TAA AGT T-3'; RAN siRNA#2 (si2), 5' -GCA ACA GTT GAG TTT CTT A-3'; and RAN siRNA#3 (si3), 5'-GGA GAT TGC AAG TGG TGT T-3'. The specific siRNA sequences against HSBP1 were: HSBP1 siRNA#1 (si1), 5' -CCA GAT CAT TGG GAG AAT T-3'; HSBP1 siRNA#2 (si2), 5'-CCA TGT CTG ACC AGA TCA T-3'; and HSBP1 siRNA#3 (si3), 5'-GAC CAG ATC ATT GGG AGA A-3'. The nonspecific scrambled siRNA sequence (siNC) was 5' GTC ATT TGA CTG GTG AAT T 3'. Cells were transfected with 60 nmol/l of siRNA duplexes using Lipofectamine2000 (Invitrogen, USA), according to the manufacturer's protocol.

### Cell proliferation assay

The Cell Counting Kit 8 (CCK-8) from Bimake (Shanghai, China) was used to detect the viability of ovarian cancer cells. A2780 Lin28A and PA-1 cells transfected with siNC/RAN-siRNA/HSBP1-siRNA were seeded into 96-well plates at a density of 2 × 10^3^ cells per well in a volume of 100 μl with 5 repetitions. After the cells were attached, 10 μl of CCK-8 reagent was added to each well, incubated at 37˚C for 2 hours, and the OD value at 450 nm was measured on the automatic microplate reader (Beckman, CA).

### Transwell migration assay

A2780 and PA-1 cells that had been transfected with siNC, siRAN or siHSBP1 for 24 hours were then trypsinized and resuspended in DMEM containing 0.1% FBS. Then 200 μl of cell suspension containing 10,000 cells was then added to the top chamber of trans-well (Corning Inc, USA) in 24-well plates and 700 μl DMEM containing 20% FBS was added to the bottom chamber. After incubation in an incubator overnight, the chamber was washed three times with PBS, then the cells in the chamber were fixed with 4% paraformaldehyde, the cells remaining in the top chamber were removed, and the cells in the bottom chamber were stained with crystal violet. Finally, the photograph was taken by Optical Microscope (Olympus, Japan).

### Apoptosis assay

A2780 Lin28A and PA-1 cells were transfected with siNC/siRAN/siHSBP1 for 48 hours, then digested with 0.25% trypsin without EDTA and washed three times with PBS. Next, according to the protocol of Annexin V-FITC/PI apoptosis detection kit (BD, USA), the cells were suspended in 500 μl of binding buffer. Then cells were stained twice with 5 μl PI and 5 μl Annexin V-FITC solution and incubated for 20 min at room temperature in the dark. The fluorescence intensity was then measured using a flow cytometer.

### Xenografted Tumor Models

In the tumor xenograft model, BALB/c nude female mice which aged 4 weeks were randomly divided into 5 groups: A2780 Lin28A cells lentivirus infection with shNC/shRAN-1/shRAN-2/shHSBP1- 1/shHSBP1-2. In each group, 1 x 10^7^ cells were individually injected subcutaneously into the left forelimb armpit of these nude mice. Two weeks after subcutaneous injection of OC cells, the length and width of the tumor were measured every three days with a micrometer, and the tumor volume was calculated using the formula V = 1/2 × L × W^2^. The mice were sacrificed 25 days later, the tumors were taken out, weighed and photographed. And all tumor tissues were fixed with 4% paraformaldehyde solution for about 36 h, then embedded in paraffin and sectioned for subsequent immunohistochemical staining experiments.

### Statistical analysis

All date statistics for qRT-PCR, CCK-8, and mouse xenograft tumor models were expressed as mean ± SEM. The correlation between RAN/HSBP1 and Lin28A expression in ovarian cancer tissues was analyzed by Spearman correlation coefficient, and the clinicopathological features of ovarian cancer tissue samples were analyzed by t-test. Statistical analysis was calculated with GraphPad Prism 6.0. The differences between groups were analyzed using the t-test or two-way ANOVA. A value of *P*<0.05 (*), <0.01 (**) or <0.001 (***) was considered statistically significant.

## Results

### Lin28A can up-regulate stem-like properties of ovarian cancer cells

**Figure 1:**
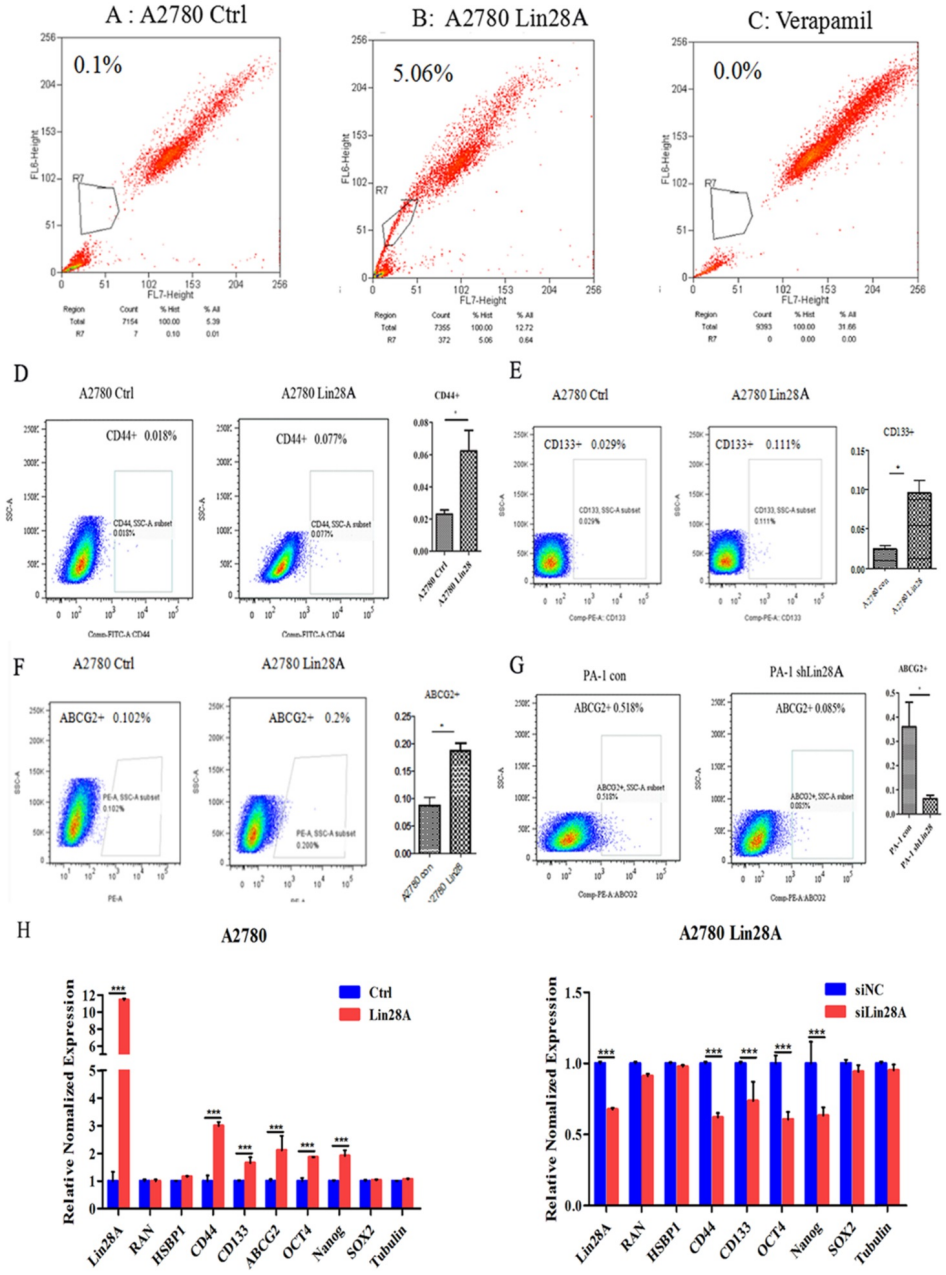
** Lin28A can up-regulate stem-like properties of OC cells. (A-C)** The effects of overexpression of Lin28A on side population cells of A2780 cells were detected by flow cytometry analysis (A2780 Ctrl and Verapamil group as negative control and blank control, respectively). **(D-G)** The role of Lin28A on the proportion of CD133^+^, CD44^+^, ABCG2^+^ cells were detected by flow cytometry analysis. **(H)** The effects of Lin28A on the expression of the stem cell marker molecules CD44, CD133, ABCG2, OCT4, Nanog and SOX2 were measured with qRT-PCR.

The results of flow cytometry analysis showed that when Lin28A was highly expressed, the side population of A2780 cells increased compared with the control group (Figure 1A-C). We then analyzed the effect of Lin28A on the proportion of OC cancer stem cell marker CD133^+^, CD44^+^, and ABCG2^+^ cells by flow cytometry. It was found that when Lin28A was highly expressed in A2780 cells, the proportion of CD44^+^, CD133^+^ and ABCG2^+^ cells increased (Figure 1D-F), and when Lin28A was knocked down in PA-1 cells, the proportion of ABCG2^+^ cells decreased (Figure 1G). Meanwhile, the expression of stem cell marker molecules in OC cells was measured with qRT-PCR. The data also showed that when Lin28A was overexpressed, the expression of stem cell marker molecules CD133, CD44, ABCG2, OCT4 and Nanog were increased, while when Lin28A was knocked down, their expression decreased (Figure 1H). The above results indicate that Lin28A enhanced stem-like properties of OC cells.

### Lin28A enrich RAN/HSBP1 mRNA and up-regulate their protein expression

**Figure 2:**
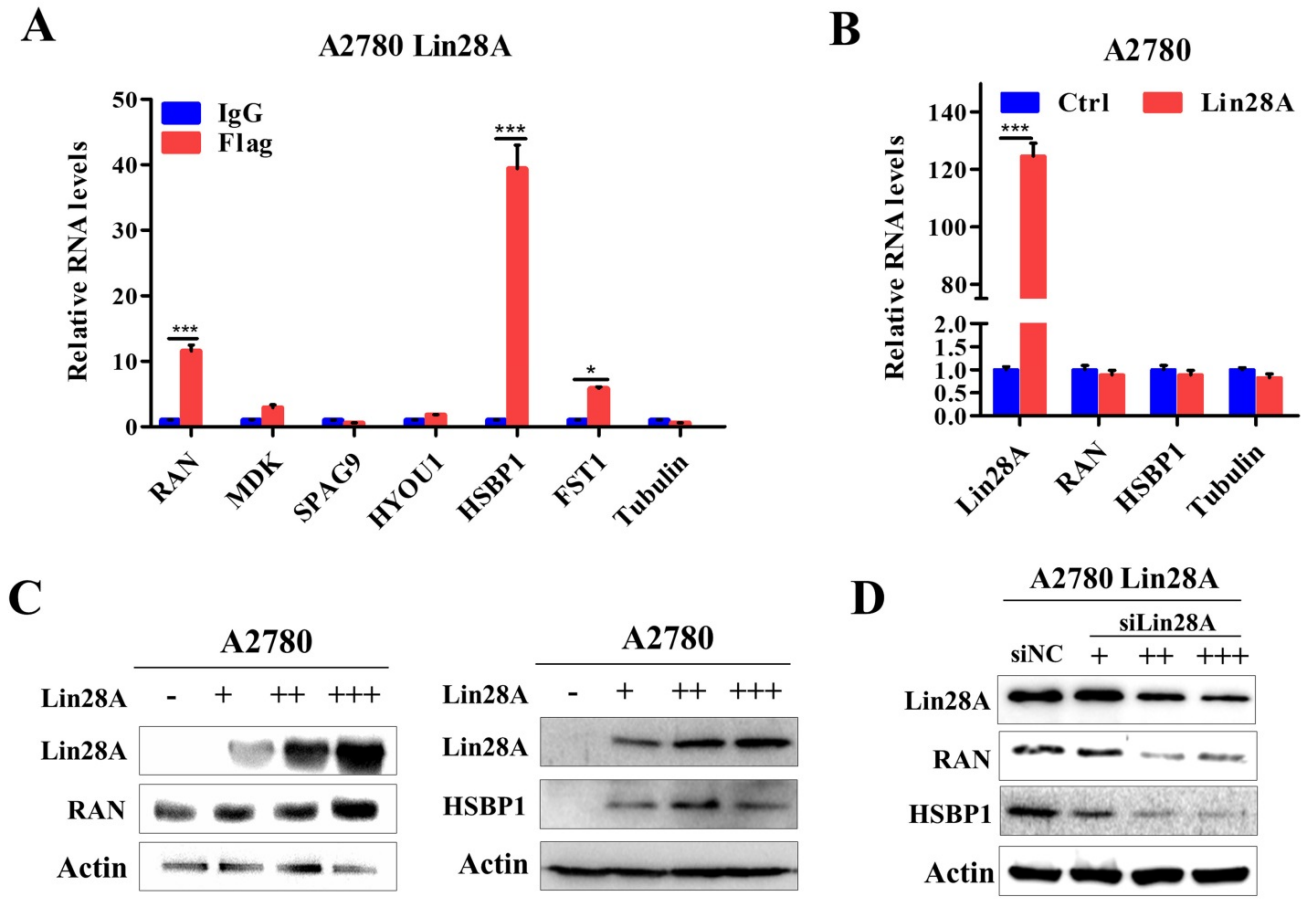
** Lin28A was bound to RAN/HSBP1 mRNA and up-regulated their protein expression. (A)** RIP assay and qRT-PCR experiments confirmed the interaction of Lin28A and the mRNAs of RAN and HSBP1. **(B)** The role of Lin28A on the RNA levels of RAN/HSBP1 was analyzed by qRT-PCR. **(C)** The effects of overexpression of different amounts of Lin28A on RAN/HSBP1 protein levels were detected with western blot, actin as an internal reference. **(D)** The effect of knockdown of different amounts of Lin28A on RAN/HSBP1 protein levels was detected with western blot, actin as an internal reference.

Our previous research have shown that when Lin28A is highly expressed, it can significantly promote the proliferation, invasion and inhibit the apoptosis of OC cells^23^, ^25^. To further explore the specific mechanism of Lin28A in OC, we found that Lin28A could enrich the mRNAs of RAN and HSBP1 by RNA immunoprecipitation (RIP) assay and qRT-PCR (Figure 2A). We then analyzed the effect of Lin28A on the expression of RAN/HSBP1 with western blot and qRT-PCR. The results indicated that Lin28A did not affect the RNA levels of RAN/HSBP1 (Figure 2B), while overexpression of Lin28A could up-regulate the protein level of RAN/HSBP1 in A2780 cells, when knocking down the expression of Lin28A, the protein levels of RAN/HSBP1 was down-regulated in A2780 Lin28A cells (Figure 2C and 2D), and the effect was dose dependent.

### Lin28A, RAN and HSBP1 are highly expressed and Lin28A level was associated with RAN/HSBP1 in OC tissues

**Figure 3:**
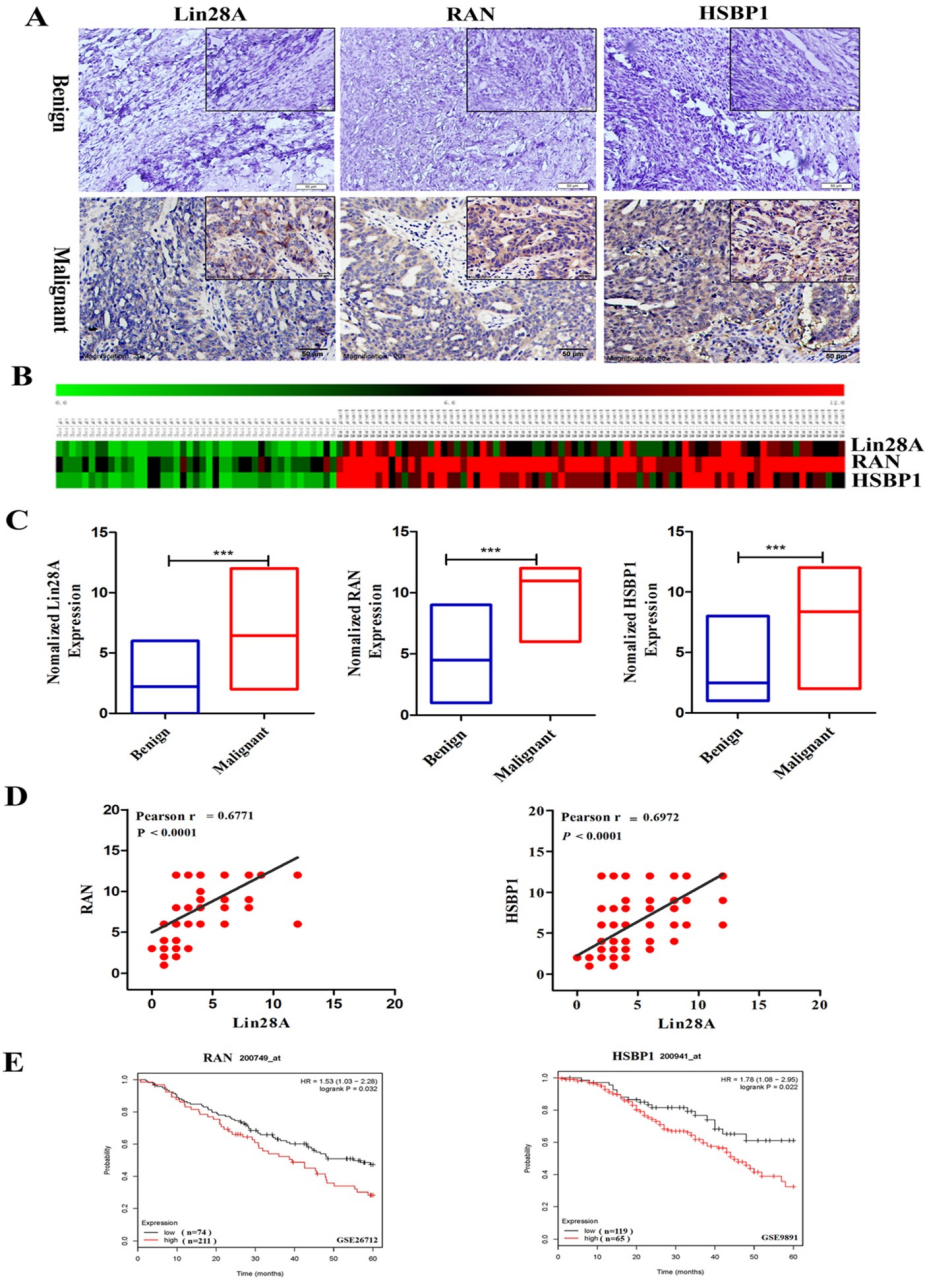
** Lin28A, RAN and HSBP1 were highly expressed and associated with poor prognosis in OC. (A)** Representative images for the expression of Lin28A and RAN/HSBP1 were shown in benign ovarian tumor tissues and malignant OC tissues by immunohistochemistry. **(B-C)** Heat maps and graphs of immunohistochemical scores based on the expression of Lin28A, RAN and HSBP1 in benign ovarian tumor tissues and malignant OC tissues. **(D)** Pearson correlation analysis indicated that the expression of Lin28A was significantly positively related to RAN (left) and HSBP1 (right) in ovarian cancer (Pearson r=0.6771/ Pearson r=0.6972, P<0.0001). **(E)** Five-year survival analysis of GEO database (GSE26712 and GSE9891) suggested the expression of RAN (left) and HSBP1 (right) were negatively associated with the prognosis of ovarian cancer patients.

Then we detected the expression of Lin28A, RAN and HSBP1 in 47 ovarian benign tumor tissues and 96 ovarian malignant tumor tissues by immunohistochemical analysis. Representative photographs revealed that Lin28A, RAN and HSBP1 are highly expressed in malignant OC, and the expression of RAN/HSBP1 is enhanced as the expression of Lin28A is increased in OC tissues (Figure 3A). Heat maps and charts based on the scores of immunohistochemistry also showed the same results (Figure 3B and 3C). Finally, the correlation analysis indicated that there was a significant positive correlation between the expression of Lin28A and RAN/HSBP1 (Pearson r = 0.6771/Pearson r = 0.6972, *P*<0.0001) (Figure 3D).

### The expression of RAN and HSBP1 are associated with survival time of OC patients

We also analyzed the GEO database (GSE26712 and GSE9891) and indicated that OC patients with highly expressed RAN or HSBP1 had a lower 5-year survival rate than those with lowly expressed RAN or HSBP1, which suggested that the expression of RAN or HSBP1 is negatively correlated with the prognosis of patients with OC (Figure 3E).

### Knockdown RAN/HSBP1 inhibited the stem-like features and the survival of OC cells

**Figure 4:**
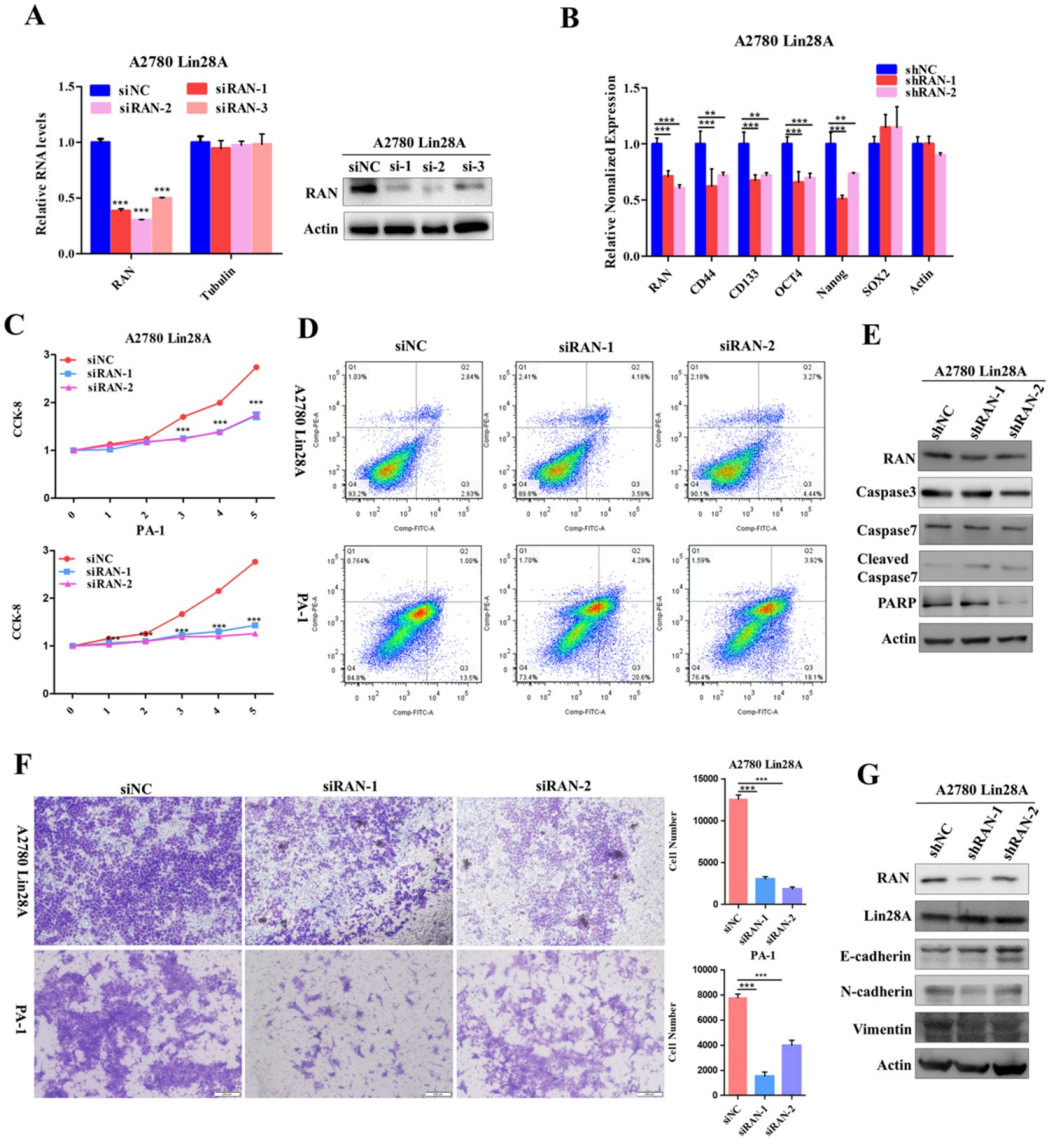
** Lin28A promoted the proliferation and invasion of OC cells by up-regulating RAN. (A)** Transiently transfected siRNA knocked down the expression of RAN at the protein level (right) and the RNA level (left) in A2780 Lin28A cells. **(B)** The role of knockdown RAN on the expression of the stem cell marker molecules CD44, CD133, ABCG2, OCT4, Nanog and SOX2 were measured with qRT-PCR. **(C)** CCK-8 analysis showed that siRAN reduced the proliferation rate of A2780 Lin28A (top) and PA-1 (bottom) cells compared to control siNC. **(D)** Knockdown of RAN by siRNA promoted the apoptosis of A2780 Lin28A and PA-1 cells by flow cytometry analysis. **(E)** Knockdown of RAN by siRNA promoted the expression of cleavage caspase-7, and reduced the expression of PARP with western blot, actin as an internal reference. **(F)** Knockdown of RAN decreased the invasion of A2780 Lin28A (top) and PA-1 (bottom) cells. **(G)** The role of knockdown RAN in the expression of invasion-related molecules was examined with western blot, actin as an internal reference.

**Figure 5:**
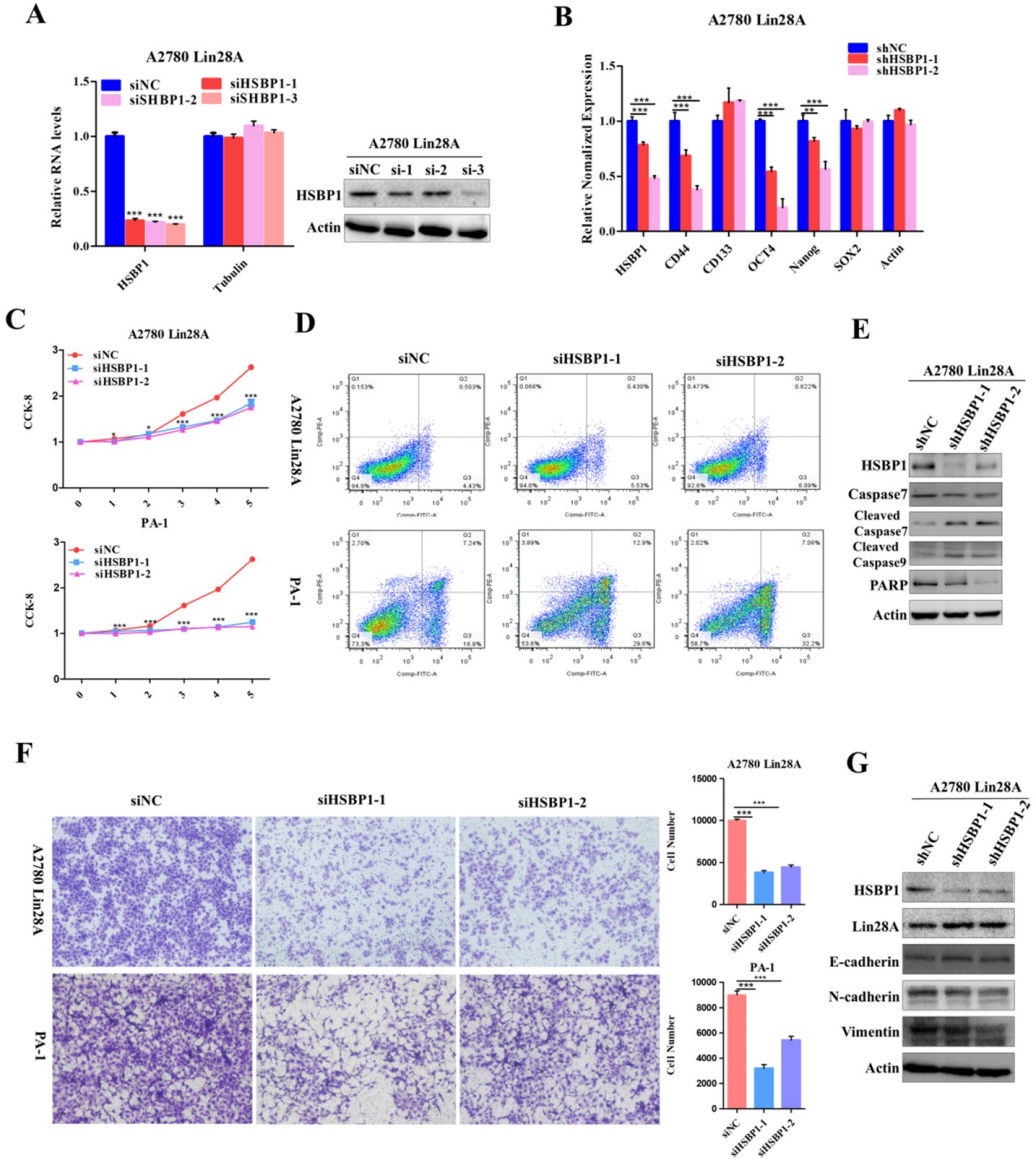
** Lin28A promoted the proliferation and invasion of OC cells by up-regulating HSBP1. (A)** Transiently transfected siRNA knocked down the expression of HSBP1 at the protein level (right) and the RNA level (left) in A2780 Lin28A cells. **(B)** The role of knockdown HSBP1 in the expression of the stem cell marker molecules CD44, CD133, ABCG2, OCT4, Nanog and SOX2 were measured with qRT-PCR. **(C)** CCK-8 analysis showed that siHSBP1 reduced the the proliferation rate of A2780 Lin28A (top) and PA-1 (bottom) cells, respectively, compared to the control siNC. **(D)** Knockdown of HSBP1 by siRNA promoted the apoptosis of A2780 Lin28A and PA-1 cells through flow cytometry analysis. **(E)** Knockdown of HSBP1 by siRNA promoted the expression of cleavage of caspase-7 and -9, reduced the expression of PARP with western blot, actin as an internal reference. **(F)** Knockdown of HSBP1 reduced the invasion of A2780 Lin28A (top) and PA-1 (bottom) cells. **(G)** The role of knockdown HSBP1 in the expression of invasion-related molecules was examined with western blot, actin as an internal reference.

Since we have found that high expression of Lin28A could up-regulate RAN/HSBP1 protein expression in OC cells. To further conform whether Lin28A promotes cell stemness, proliferation, invasion and inhibition of cell apoptosis by regulating RAN/HSBP1 expression. We then investigated the role of knockdown of RAN/HSBP1 in OC cells. We designed and synthesized three specific siRNAs targeting RAN/HSBP1 and transiently transfected them into PA-1 and A2780 Lin28A cells. The protein levels and RNA levels of RAN/HSBP1 were effectively knocked down in PA-1 and A2780 Lin28A cells with western blot and qRT-PCR analysis (Figure 4A and 5A). The results of qRT-PCR indicated that the expression of stem cell marker molecules CD133, CD44, OCT4 and Nanog were decreased after knockdown of RAN in A2780 Lin28A cells that with high expression of Lin28A (Figure 4B). While when HSBP1 was knocked down, the expression of stem cell marker molecules CD44, OCT4 and Nanog were decreased (Figure 5B). The CCK-8 assay data indicated that the knockdown of RAN/HSBP1 significantly decreased the cell viability of PA-1 and A2780 Lin28A cells (Fig 4C and 5C). At the same time, we also detected the effect of RAN/HSBP1 on apoptosis of OC cells. When RAN/HSBP1 was knocked down, the apoptosis of A2780 Lin28A and PA-1 cells increased significantly compared with siNC group by flow cytometry analysis (Figure 4D and 5D). To further verify this result, we inspected the effects of knockdown of RAN/HSBP1 on the expression of apoptosis-related molecules with western blot. The results suggested that when RAN/HSBP1 was knocked down, the protein expression of cleaved caspase-7 was increased and the expression of DNA damage repair protein PARP was decreased compared with the siNC group (Figure 4E and 5E). This further confirmed that knockdown of RAN/HSBP1 inhibited the stemness and survival of OC cells by down-regulating the stem marker and inducing cell apoptosis, and also suggested that Lin28A can promote the stem-like characteristics and induce the apoptosis of OC cells by up-regulating the expression of RAN/HSBP1.

### Knockdown RAN/HSBP1 inhibited the invasion of OC cells

The siRNAs against RAN/HSBP1 were transfected into A2780 Lin28A and PA-1 cells for 48h to evaluate the effect of RAN/HSBP1 on the invasion ability of OC cells. The pictures showed a significant reduction in the number of cells passing through the chamber in the A2780 Lin28A/siRAN group and the PA-1/siRAN group compared to the siNC group (Fig. 4F), indicating that knockdown of RAN can restrain the invasion ability of OC cells. Similarly, when HSBP1 was knocked down, the number of A2780 Lin28A cells and PA-1 cells passing through the chamber was also significantly reduced (Fig. 5F). To further explore its mechanism of invasion, several EMT-related molecules were analyzed with western blot. The results indicated that when RAN or HSBP1 was knocked down, the expression of Vimentin and N-cadherin which promoted cell invasion were down-regulated, and the expression of E-cadherin that inhibited cell invasion was increased (Figure 4G and 5G). The above evidences suggested that Lin28A promoted the invasion ability of OC cells by up-regulating the expression of RAN and HSBP1.

### Lin28A promoted the tumor growth of OC xenograft by upregulating RNA/HSBP1 *in vivo*

**Figure 6:**
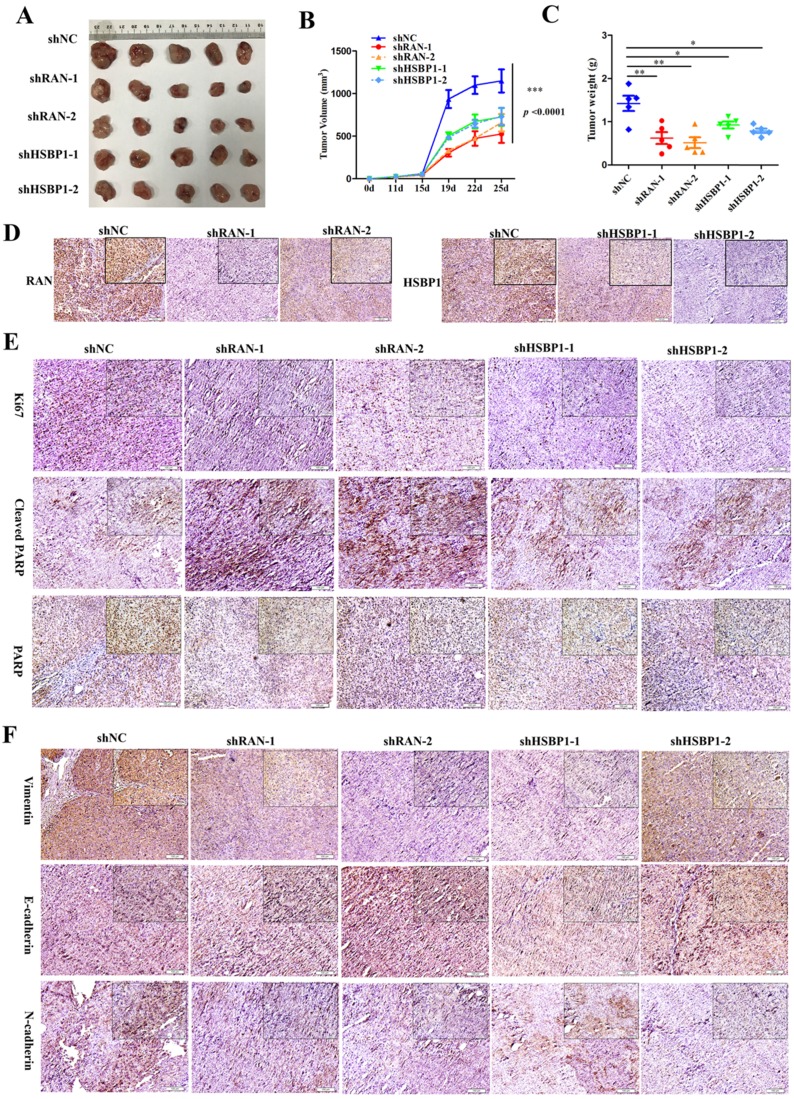
** Lin28A increased the tumor growth and invasion of OC xenograft by up-regulating RNA/HSBP1 *in vivo*. (A)** The photos of stripped tumor from shNC, shRAN (#1 and #2) and shHSBP1 (#1 and #2) groups (n=5). **(B)** The tumor growth curve of shNC, shRAN (#1 and #2) and shHSBP1 (#1 and #2) groups. **(C)** The final tumor weight of shNC, shRAN (#1 and #2) and shHSBP1 (#1 and #2) groups. **(D)** Paraffin sections were stained with anti-RAN, anti-HSBP1 and anti-Ki67 antibodies. **(E)** Representative pictures of paraffin sections were stained with anti-Ki67, anti-cleaved PARP and anti-PARP antibodies. **(F)** Representative pictures of paraffin sections stained with anti-vimentin, anti-N-cadherin and anti-E-cadherin antibodies.

Although we have recognized the important role of RAN and HSBP1 in OC cells and OC tissues, we further investigated whether they have the same effect *in vivo*. To examined the effect of RAN/HSBP1 on the tumor formation of OC cells, we transplanted A2780 Lin28A shNC, shRAN (#1 and #2) and shHSBP1 (#1 and #2) cells into the Balb/c nude mice to form tumor, separately. After the tumor grew, the length and width of the subcutaneous tumor were measured every three days, and the mice were killed on the 25th day after the initial injection of the cells. Images of tumors that excised subcutaneously from mice showed that tumors in the shNC group were larger than those in the shRAN (#1 and #2) and shHSBP1 (#1 and #2) groups (Figure 6A ), and statistical results of tumor weight and volume also indicated that knockdown of RAN/ HSBP1 inhibited the growth of OC xenograft *in vivo* (Figure 6B and 6C). The images of IHC staining suggested that tumor tissues of shRAN (#1 and #2) and shHSBP1(#1 and #2) group had a lower expression levels of RNA and HSBP1 compared with the control group A2780 Lin28A shNC group (Figure 6D). The immunohistochemistry results also showed that the expression levels of RAN and HSBP1 were positively correlated with Ki67, which is a cell proliferative nuclear antigen, indicating the rate of cell proliferation (Figure 6E). Representative images of immunohistochemistry indicated that the knockdown of RAN/HSBP1 promoted the expression of Cleaved PARP, while the expression of PARP decreased (Figure 6E), indicating that knockdown of RAN or HSBP1 can promote OC cells apoptosis *in vivo*. Meanwhile, the results of immunohistochemistry confirmed that shRAN or shHSBP1 caused the down-regulation of Vimentin, N-cadherin, and promoted the expression of E-cadherin (Figure 6F), indicating that the knockdown of RAN and HSBP1 inhibited the invasive ability of ovarian tumor *in vivo*.

## Discussion

In the previous study, we found that Lin28A could also play a role in promoting the development of malignant OC by up-regulating the expression of the interacting protein ROCK2^25^, in addition to the regulation of let-7^27^. As a pluripotent stem factor, Lin28A plays a significant role in biological activities, and the functional mechanism is also extremely complicated^28^-^32^. In this study, we also found that Lin28A interacts with RAN and HSBP1 mRNA, and Lin28A up-regulated its protein expression. Previous studies have shown that Lin28A can recruit RHA helicase (RHA) to the polysomes to increase the translation levels of its binding target mRNA^33^. We confirmed that Lin28A can bind to the mRNA of RAN/HSBP1 by RIP assay, suggesting that Lin28A may promote the protein synthesis of RAN/HSBP1 as it doesn't affect the mRNA level of RAN/HSBP1 but affect the translation of its protein. We infer that Lin28A may up-regulated the protein expression of RAN/HSBP1 through similar mechanism as well as Oct4^33^.

RAN is a small ras-associated GTPase that plays an important role in nuclear transport, cell mitosis and nuclear envelope formation, and is thought to have different roles in a range of cell functions^34^-^37^. Studies have shown that abnormal expression of RAN and subsequent genetic instability are associated with cancer progression^38^-^40^. It has been reported that inhibited the expression of RAN could cause abnormal mitotic spindle formation, mitochondrial dysfunction and apoptosis in several cancer cell lines^41^, ^42^. And RAN has been found to be highly expressed in a variety of cancers, and it has been demonstrated that overexpression of RAN can promote the invasive ability of cancer cells^43^-^45^. However, the function and mechanism of RAN in OC remains largely unknown.

**Figure 7:**
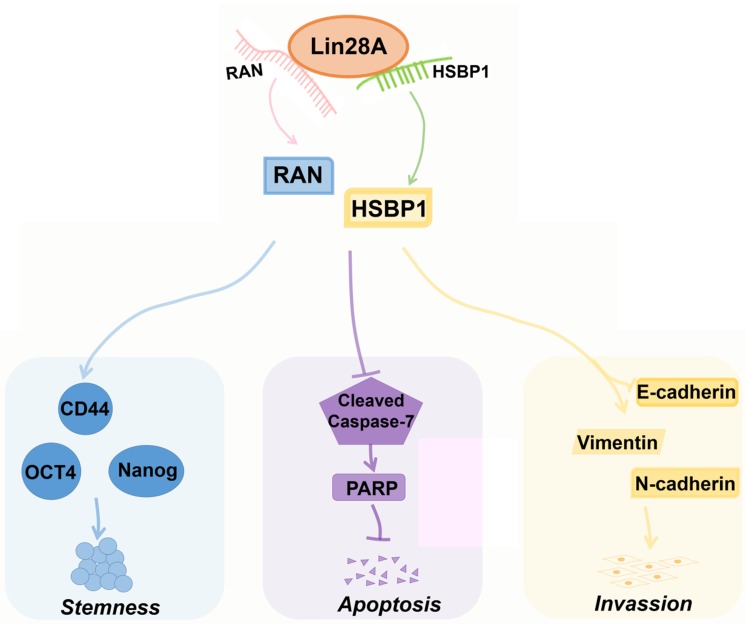
** A model for Lin28A/RAN/HSBP1 promotes stemness and invasion and inhibits cell apoptosis of OC cells.** Lin28A enhanced the stem-like features through up-regulating RAN/HSBP1 and subsequent surface marker molecules CD44, OCT4 and Nanog; Lin28A inhibited the apoptosis of OC cells through up-regulating RAN/HSBP1 and subsequent inhibition of poly ADP ribose polymerase PARP; Lin28A promoted the invasion of OC cells by up-regulating RAN/HSBP1 and subsequent expression of EMT-related molecule.

HSBP1 is an evolutionarily highly conserved heat shock factor binding protein that directly binds to the DNA of heat shock factor 1 (Hsf1) and inhibits its transcriptional activity^46^, ^47^. HSBP1 contains a heptad-repeat in the primary sequence and is believed to maintain a trimer form in solution^48^, ^49^. HSBP1 is composed of 76 amino acid residues and is a small protein (<10 kDa)^49^. There is a long α-helix in the HSBP1 protein structure with unstructured amino (N)-and carboxyl (C)-terminal regions^50^. Currently, a study only pointed out that HSBP1 played a role in the development of early embryos^51^. However, the function of HSBP1 is largely unknown and its association with carcinogenesis and prognosis is unclear. In this study, we further confirmed that Lin28A can up-regulate the protein expression of RAN/HSBP1 to promote the stemness, the proliferation, the invasion and to inhibit the apoptosis of ovarian cancer cells (Figure 7). Lin28A promoted the expression of stem molecules CD44, OCT4 and Nanog by up-regulating the protein expression of RAN/HSBP1 to promote the stemness of OC cells (Figure 7). Lin28A also inhibited the apoptosis of OC cells by up-regulating the protein expression of RAN/HSBP1 and then the expression of PARP (Figure 7). Lin28A also played a role in promoting the invasion of OC cells by up-regulating RAN/HSBP1 and EMT-related molecules (Figure 7). Our results demonstrated that Lin28A, RAN and HSBP1 were highly expressed and the expression of Lin28A was associated with that of RAN and HSBP1 in OC tissues, and their high expression is negatively correlated with the prognosis of OC patients.

## Conclusion

In conclusion, our study suggest that highly expressed Lin28A/RAN/HSBP1 can be used as diagnostic and prognostic markers in patients with OC, whereas RAN/HSBP1 may be a potential new target for gene therapy of OC.

## Supplementary Material

Supplementary table S1.

## Abbreviations

OC: ovarian cancer
RAN: ras-related nuclear protein
HSBP1: heat shock factor binding protein 1
RIP: RNA immuno-precipitation
qRT-PCR: Quantitative real-time PCR
siRNAs: small interference RNAs
Hsf1: heat shock factor 1
