# Supplementary material for: Lin28A Regulates Stem-like Properties of Ovarian Cancer Cells by Enriching RAN and HSBP1 mRNA and Up-regulating its Protein Expression
Source: Int J Biol Sci. 2020 Apr 15;16(11):1941–53. doi: 10.7150/ijbs.43504 (PMC7211169; doi:10.7150/ijbs.43504)
Supplement: Supplementary file 1 — Supplementary table S1. [file ijbsv16p1941s1.pdf]

Table S1

Primer sequences used for qRT- PCR

Gene	Forward primer sequence (5′–**3′**)	Reverse primer sequence (5′–**3′)**
RAN	GAAAGTGAAGGCGAAATCCA	CAAGTTAGGGTCTCCAATGAGC
ABCG2	CGCACAGAGCAAAGCCATTT	ACCAGGTTTCATGATCCCATTGA
NANOG	AATGGTGTGACGCAGGGATG	CTATAGCCAGAGACGGCAGC
CD133	ATCCTTTCCATTACGGCGGC	CTCAAGGCACCATCCCGTG
OCT4	GCTGGATGTCAGGGCTCTTT	CTCGGACCACATCCTTCTCG
SOX2	ATGGACAGTTACGCGCACAT	TTCCTGCAAAGCTCCTACCG
HSBP1	CCTGCARCAGATGCAAGATA	TTGGAAAAATGCCAGATTCC
CD44	AAGGTGGAGCAAACAACC	AGCTTTTTCTGCCCACA
Lin28A	CGGGCATCTGTAAGTGGTTC	CAGACCCTTGGCTGACTTCT
Actin	CCTATCGAGCATGGAGTGGT	CTGAGGCATAGAGGGACAGC
